# Integrated analysis of stem cell-related genes shared between type 2 diabetes mellitus and sepsis

**DOI:** 10.3389/fchem.2025.1666651

**Published:** 2025-09-19

**Authors:** Yubo Wang, Xinhai Jiang, Xiaohan Song, Zhijie Ma, Yan Wang

**Affiliations:** 1 Department of Pharmacy, Beijing Ditan Hospital, Capital Medical University, Beijing, China; 2 Phase I Clinical Trial Unit, Beijing Ditan Hospital, Capital Medical University, Beijing, China; 3 Beijing Key Laboratory of New Drug Mechanisms and Pharmacological Evaluation Study, Institute of Materia Medica, Chinese Academy of Medical Sciences and Peking Union Medical College, Beijing, China; 4 College of Agroforestry and Medicine, The Open University of China, Beijing, China

**Keywords:** type 2 diabetes mellitus, sepsis, stem cells, biomarkers, machine learning

## Abstract

**Background:**

Diabetes-induced immune impairment and insulin resistance increase infection risk, which may progress to sepsis that further deteriorates diabetic status. Stem cell-based interventions show therapeutic potential for both diseases. This study sought to uncover common stem cell-related genes (SCRGs) between T2DM and sepsis.

**Methods:**

The GSE15932 dataset for T2DM and GSE65682 dataset for sepsis from the Gene Expression Omnibus (GEO) were utilized to locate common differentially expressed genes (DEGs), which were then intersected with SCRGs to derive shared differentially expressed SCRGs (DE-SCRGs). The stem-cell-related biomarkers were discovered through combining functional similarity analysis, machine learning algorithms, and receiver operating characteristic (ROC) curves. Subsequently, functional enrichment analysis, immune infiltration, and single-cell analyses were conducted to investigate the potential pathways by which biomarkers regulate T2DM and sepsis. Finally, the expression of biomarkers was further verified at both transcriptional and protein levels through the establishment of an *in vitro* model of T2DM-sepsis.

**Results:**

Through a comprehensive analysis, CAPG and DDAH2 were found and those were significantly highly expressed in both T2DM and sepsis. Analysis of functional enrichment demonstrated they were implicated in “FC gamma R-mediated phagocytosis” and “Ribosome”. Immune infiltration indicated a considerable disparity in the quantity of CD8 T cells and monocytes when comparing T2DM versus control groups, as well as sepsis versus control groups. At the cellular level, notable differences in CARG expression among alpha cells, beta cells, delta cells, and pancreatic stellate cells (PSCs) were observed in the two groups being compared. At transcription and protein levels, CAPG and DDAH2 were significantly more highly expressed in the T2DM-sepsis model than in the controls. The results corroborated the bioinformatics analysis conclusions, reinforcing the study’s validity.

**Conclusion:**

Two common stem cell-related biomarkers (CAPG and DDAH2) and their common pathways between T2DM and sepsis were discovered, providing new insights for further molecular mechanism studies.

## 1 Introduction

Diabetes and its complications, given their widespread prevalence, represent a significant hazard to global health systems. The worldwide prevalence of diabetes has increased fourfold in the past 3 decades, with growing urbanization intensifying this change (Gassasse et al., 2017; Thanikachalam et al., 2019). An estimated 463 million adults (9.3%) aged 20–79 years worldwide have diabetes, with projections indicating this number will increase to 700 million by 2045 (Saeedi et al., 2019). It is estimated that from 2021 to 2024, the growing population with the disease in Southeast Asia alone reaches about 60 million people (Rudd et al., 2020). Over 90% of diabetes cases have type 2 diabetes mellitus (T2DM), a metabolic disorder defined by insulin resistance and a predisposition to microvascular complications (such as diabetic eye and kidney diseases) and macrovascular complications (including cardiovascular and cerebrovascular events) (Chiu and Legrand, 2021; Gassasse et al., 2017; Holman et al., 2015; Saeedi et al., 2019; Thanikachalam et al., 2019). Although gastrointestinal weight-loss surgery and organ transplantation are now viable procedures, the former demonstrates limited long-term efficacy, while the latter is restricted by patient age and organ donor availability (Font et al., 2020). Consequently, comprehending the molecular alterations in T2DM is pivotal for developing preventive strategies and improving treatments.

Sepsis, a life-threatening syndrome of organ dysfunction, occurs when the host’s response to infection becomes dysregulated (Chiu and Legrand, 2021). Globally, there are more than 50 million new cases of sepsis each year, and the number of deaths reaches 11 million, accounting for 1/5 of the global deaths (Rudd et al., 2020). In China, the number of sepsis patients dying each year is as high as one million and is on the rise year by year. The latest data show that the mortality rate of sepsis in Europe and America has reached 26%, and the mortality rate of its severe stage (infectious shock) is even as high as 40% (Rudd et al., 2020). In China, patients with sepsis account for about 20.6% of the hospitalizations in intensive care units, and sepsis and infectious shock exhibit the death rates of 35.5% and 53.3%, respectively, imposing significant strain on healthcare resources (Font et al., 2020). In addition, the diverse manifestations of sepsis and the restricted effectiveness of antibiotics, fluid resuscitation, and organ-supportive therapies present a considerable clinical diagnostic and therapeutic challenge (Huang et al., 2019; Liu et al., 2022). Therefore, the study of the pathophysiological mechanisms of sepsis and the exploration of specific therapeutic approaches are of great research value.

Diabetes and sepsis are closely interconnected pathologically, with each condition exacerbating the other in a detrimental cycle that hastens disease progression (Frydrych et al., 2018; Trevelin et al., 2017). Patients with diabetes are susceptible to sepsis, whereas those with sepsis frequently have aberrant blood glucose levels and may subsequently develop diabetes. Considering the limitations of traditional therapy, stem cell-based therapeutics provide significant potential for the management of T2DM and sepsis. Stem cells, particularly mesenchymal stem cells (MSCs), have exhibited the capacity to ameliorate insulin resistance, β-cell malfunction, and tissue damage, while also fostering tissue homeostasis and glycemic regulation for T2DM (Hoang et al., 2022; Mikłosz and Chabowski, 2023; Zang et al., 2022). Their synergistic interaction with adjunctive therapy modalities enhances their clinical efficacy (Saha et al., 2023). Additionally, MSCs are of significant interest in sepsis therapy due to their ease of isolation and proliferation (Keane et al., 2017). Therefore, research focusing on stem cells and their related genes is crucial for elucidating the regulatory mechanisms of T2DM and sepsis, as well as for enhancing treatment development.

In this study, utilizing transcriptomic data from the Gene Expression Omnibus (GEO) collection, conserved stem cell-related biomarkers for T2DM and sepsis were found by differential expression analysis, machine learning approaches, and evaluation of expression in datasets (prospective study). Subsequently, functional enrichment, immune infiltration, drug prediction, and single-cell analysis were performed to further elucidate the potential molecular mechanisms by which these biomarkers govern disease progression. Ultimately, the expression of identified biomarkers was confirmed using *in vitro* tests. This work not only enhances our comprehension of mechanisms underlying T2DM and sepsis but also offer novel references for illness diagnosis and therapeutic development.

## 2 Materials and methods

### 2.1 Data source

Following database searching, target microarray datasets were obtained from the GEO database (https://www.ncbi.nlm.nih.gov/geo/) using the keywords “Type 2 diabetes mellitus” and “Sepsis.” For T2DM, GSE15932 and GSE20966 were chosen. For sepsis, GSE65682 and GSE95233 were included in our study, with another one T2DM-related single-cell RNA sequencing (RNA-seq) dataset, GSE195986 (Table 1). All GEO datasets were analyzed using the preprocessed data provided by the database, which had undergone standard normalization, filtering, and necessary batch correction according to the official GEO pipelines. Additionally, an overall of 26 stem gene sets were sourced from the StemChecker database (http://stemchecker.sysbiolab.eu/), and 4,419 stem cell-related genes (SCRGs) were obtained after integration.

**TABLE 1:** Details of GEO datasets used in this study.

Disease	GEO series	Platform	Case samples	Control samples	Group	Source	Cohort characteristics	Missing data
T2DM	GSE15932	GPL570	8	8	Training cohort	Peripheral blood	Small cohort, peripheral blood from T2DM vs. healthy controls	Minimal (metadata complete)
	GSE20966	GPL1352	10	10	Validation cohort	pancreas	Moderate cohort, pancreatic tissues, paired case-control	Minimal (metadata complete)
	GSE195986	GPL16791	7	4	Single-cell dataset	pancreatic islet	Single-cell dataset, pancreatic islets	Possible dropout cells (scRNA QC applied)
Sepsis	GSE65682	GPL13667	760	42	Training cohort	Whole blood	Large multicenter sepsis cohort, whole blood, unbalanced case-control	Some missing clinical covariates reported in GEO
	GSE95233	GPL570	102	22	Validation cohort	Whole blood	Independent validation cohort, whole blood	Minor missing values in metadata

### 2.2 Differential expression analysis

The “limma” R package (version 3.54.2) was utilized to investigate differentially expressed genes (DEGs) between T2DM and control groups in GSE15932 with |log fold change| (|logFC|) > 0.5 and *P*-value <0.05 as screening thresholds (Ritchie et al., 2015). The DEGs between sepsis and control groups in GSE65682 were identified based on |logFC| > 1 and *P*-value <0.05. The results were displayed by volcano plots drawn utilizing the “ggplot2” R package (version 3.5.1). Moreover, heatmaps were plotted by the “pheatmap” R tool (version 1.0.12) to show the top 20 upregulated and top 20 downregulated genes. Subsequently, the results of these datasets were intersected with SCRGs to acquire the shared differentially expressed SCRGs (DE-SCRGs) among these two datasets, with all reported *P*-values adjusted for false discovery rate (FDR) (Supplementary Table S1).

### 2.3 Functional enrichment analysis

The molecular mechanisms of shared DE-SCRGs of T2DM and sepsis were identified integrating Gene Ontology (GO) and Kyoto Encyclopedia of Genes and Genomes (KEGG) via the “clusterProfiler” R package (version 4.6.2) (Wu et al., 2021). A GO item or KEGG pathway was considered statistically significant when annotating DEGs with a *P* < 0.05 threshold.

### 2.4 Identification of shared stem cell-related biomarkers for T2DM and sepsis

Firstly, the similarities in the function of these shared DE-SCRGs were investigated via the “GOSemSim” R package (version 2.30.2), and the shared DE-SCRGs with similar functions were enrolled in the following study. Combined with the annotation information of biological processes (BP), cellular components (CC), and molecular functions (MF) in the GO database, a composite score was derived by calculating the geometric mean of the genes in MF, BP, and CC, which reflects the molecular function, biological process, and cellular localization information of the genes, thus identifying the genes in the group that interact most strongly with other genes. Then, to minimize bias in the diagnostic models, we used two machine learning algorithms to locate characteristic genes, namely, least absolute shrinkage and selection operator (LASSO) (using the “glmnet” R package (version 4.1–8)) (Friedman et al., 2010) and Boruta (using the “Boruta” R package (version 8.0.0)). The final predicted candidate genes in sepsis complicated with T2DM were defined as the intersection of predictions from the two different machine learning algorithms. Thereafter, the expression of these candidate genes in GSE15932, GSE20966, GSE65682, and GSE95233 was analyzed, and the diagnostic efficacy of genes exhibiting differential expression between disease and control groups, along with consistent expression patterns, was assessed using receiver operating characteristic (ROC) curves. The area under the curve (AUC) greater than 0.7 indicated a good diagnostic efficacy. Therefore, the genes with AUC >0.7 were defined as biomarkers.

### 2.5 Construction and assessment of the nomogram for T2DM and sepsis

To measure the predictive effectiveness of biomarkers for T2DM and sepsis, the nomogram predictive model was constructed based on biomarkers expression using the “rms” R function (version 6.8–0). Moreover, to determine their effectiveness, calibration curves were generated to assess the nomogram.

### 2.6 Localization analysis

To understand the exact location of the biomarker on the chromosome, the chromosome localization analysis was employed by the “RCircos” R package (version 1.2.1) (Zhang et al., 2013). Furthermore, the FASTA sequences of biomarkers have been extracted from the database maintained by NCBI (https://www.ncbi.nlm.nih.gov/gene), and according to these sequences, the mRNALocater repository (http://bio-bigdata.cn/mRNALocater/) was applied for subcellular localization analysis to understand its spatial distribution within the cell.

### 2.7 Gene set enrichment analysis (GSEA) and gene set variation analysis (GSVA)

To investigate potential pathways in which biomarkers were involved and the pathways that were activated or inhibited during disease progression, GSEA (“clusterProfiler”) and GSVA was carried out. The “c2. cp.kegg_medicus.v2023.2. Hs.symbols.gmt” was retrieved from the MSigdb directory (http://www.gsea-MSigdb.org/gsea/msigdb) as the reference set. The GSEA pathways were considered statistically significant with |NES| > 1 and adjusted *P*-value <0.05 as thresholds. The GSVA score of each pathway was calculated with the “GSVA” R package (version 1.46.0) (Hänzelmann et al., 2013), while the disparities between disease and control groups were measured by the “limma” R package (version 3.54.2). The pathways were considered significant differences with |t| > 2 and *P*-value <0.05.

### 2.8 Immune infiltration analysis

To figure out the 22 immune cells infiltration dynamics in diseases (T2DM or sepsis) and control groups, the CIBERSORT algorithm (version 0.1.0) was applied to estimate the proportion of each immune cell subsets infiltration. The disparities in infiltration levels between disease and control groups were analyzed via the Wilcoxon test (*P*-value <0.05). Subsequently, the associations between biomarkers and immune cells counts were assessed employing the Spearman rank correlation.

### 2.9 Construction of regulatory networks

To further understand interactions in regulatory networks, the miRNAs that regulated biomarkers were predicted utilizing the starBase database (https://rnasysu.com/encori/). Moreover, this information was also applied to forecast the lncRNAs that had regulatory relationships with miRNAs predicted (parameter settings: clipExpNum >30, clipExpNum: the number of CLIP-seq experiments supported). The ChEA3 database was applied to predict transcription factors (TFs) that had regulatory relationships with biomarkers, and TFs that were supported by ChIP-seq data in the ENCODE database were further screened. Finally, results were imported into the Cytoscape program in order to present the lncRNA-miRNA-mRNA connection and TF-mRNA structure.

### 2.10 Pharmaceutical prediction and molecular docking

To investigate possible targeted therapies for biomarkers, the medications that reacted with biomarkers were predicted through the DSIGDB database (https://dsigdb.tanlab.org/DSigDBv1.0/) (*P* < 0.05) (displaying via Cytoscape). Subsequently, the top 2 drugs according to *P*-value ranking were chosen to perform the molecular docking. The 3D structures of drugs (SDF file) were extracted from the PubChem database and were then converted to PDB files using the Babel GUI. The protein three-dimensional structures (PDB files) of biomarkers were obtained from the AlphaFold Database. The AutoDock software was employed to perform preprocessing steps, including the removal of water molecules and small molecule ligands from the protein structure, and molecular docking was conducted through AutoDock Vina. Finally, results were shown by PyMol.

### 2.11 Single-cell analysis

We implemented the “Seurat” R package (version 5.1.0) for assessing the scRNA-seq data. (Hao et al., 2024). Initially, quality control (QC) was conducted, and the screening criteria were as follow: (1) cells were retained if the gene count ranged between 200 and 2,000; (2) cells exhibiting a mitochondrial gene percentage exceeding 5% were discarded; (3) genes with 200 < count number <5,000 were preserved; and (4) genes covered by less than 3 cells were removed. After standardization, the top 2,000 via-based highly variable genes (HVG) were identified in the FindVariable Features function. Subsequently, the dimensionality reduction analysis was conducted to determine statistically significant principal components by the ScaleData and the JackStrawPlot functions. Principal component analysis (PCA) was performed using the top 2,000 HVG, the top 30 statistically significant principal components (PCs) from the PCA were chosen for subsequent analyses. Next, the cluster analysis was conducted using the FindNeghbors and FindCluster programs, and cell clusters were annotated according to the highly-variable genes in each cell cluster compared to marker genes in each cell type obtained from the published literature (Fang et al., 2019; Segerstolpe et al., 2016; Weng et al., 2023). Moreover, the proportion of each cell type in T2DM and control groups was measured. Furthermore, the expression patterns of every single biomarker in each individual cell type were examined, and variations between T2DM versus control sets were evaluated by the method known as the Wilcoxon test. Additionally, to further reveal interactions among all cell types, CellChat (version 1.6.1) was applied to conduct cellular communication analysis.

### 2.12 Cell cluster

The mouse pancreatic β cell line MIN6 were obtained from Yiaobang (Beijing) Biotechnology Research Co., Ltd. (Beijing, China), and cultured in Dulbecco’s Modified Eagle Medium (DEME) enriched with 15% fetal bovine serum (FBS), 0.05 mM β-Mercaptoethanol, and 1% penicillin-streptomycin (P/S) and maintained at 37 °C in a humidified atmosphere containing 5% CO_2_. To obtain an *in vitro* T2DM-sepsis model, MIN6 cells were initially deprived of nutrients using 0.5 percent FBS for a total of 24 h, then induced with 35 mM glucose for 24 h, and next induced with 1 μg/mL LPS for 24 h (Amyot et al., 2012).

### 2.13 Real-time quantitative polymerase chain reaction (RT-qPCR)

Total RNA was extracted from MIN6 cells with the FastPure Complex Tissue/Cell Total RNA Isolation Kit (RC113-01, Vazyme, China) following the manufacturer’s guidelines. Moreover, the Nano-500 Micro-Spectrophotometer was applied to examine the purity of RNA. The ABScript Ⅲ RT Master Mix for RT-qPCR with gDNA Remover (RK20429, ABclonal, China) was utilized for the reverse transcription of RNA into cDNA. The RT-qPCR procedure was used to assess biomarkers generation by the Genious 2X SYBR Green Fast RT-qPCR Mix (RK21205, ABclonal, China). With GAPDH as the internal reference, the 2^−ΔΔct^ technique was utilized for measuring the gene expression. The normalized primers employed were GAPDH: Forward (F): 5′-AGG​TCG​GTG​TGA​ACG​GAT​TTG-3′, Reverse (R): 5′-TGT​AGA​CCA​TGT​AGT​TGA​GGT​CA-3’; CAPG: F: 5′-TGC​CCA​TAG​CAC​GAG​AGA​G-3’; R: 5′-TCA​TTG​CCT​TGA​ACC​TCA​CGG-3’; and DDAH2: F: 5′-GGA​CCT​GGC​TAA​AGC​TCA​AAG-3’; R: 5′-CAG​GGC​CTT​GTG​ATT​AGG​GC-3’.

### 2.14 Western blotting (WB)

Total protein from MIN6 cells was extracted utilizing radioimmunoprecipitation assay (RIPA) lysis buffer supplemented with 100× PSMF protease inhibitor. The protein content was confirmed by the BCA Protein Assay Kit (P0010, Beyotime Biotechnology, China). Subsequently, using sodium dodecyl sulfate-polyacrylamide gel electrophoresis (SDS-PAGE), protein was separated and transferred onto PVDF membranes (0,000,279,048, Millipore, American). Following the blocking procedure with 5% milk that had been skimmed, the membranes were then treated with primary antibodies overnight at 4 °C. After incubation with secondary antibodies, the membranes were developed using a chemiluminescence detection reagent. Protein bands have been calculated with ImageJ software.

### 2.15 Statistical analysis

All statistical analyses were carried out using the R program and GraphPad Prism 8.0. The disparities were analyzed via the Wilcoxon test (n = 2) and Student’s t-test. A meaningful *P*-value is below 0.05.

## 3 Results

### 3.1 Identification and enrichment analysis of shared DE-SCRGs in T2DM and sepsis

**FIGURE 1:**
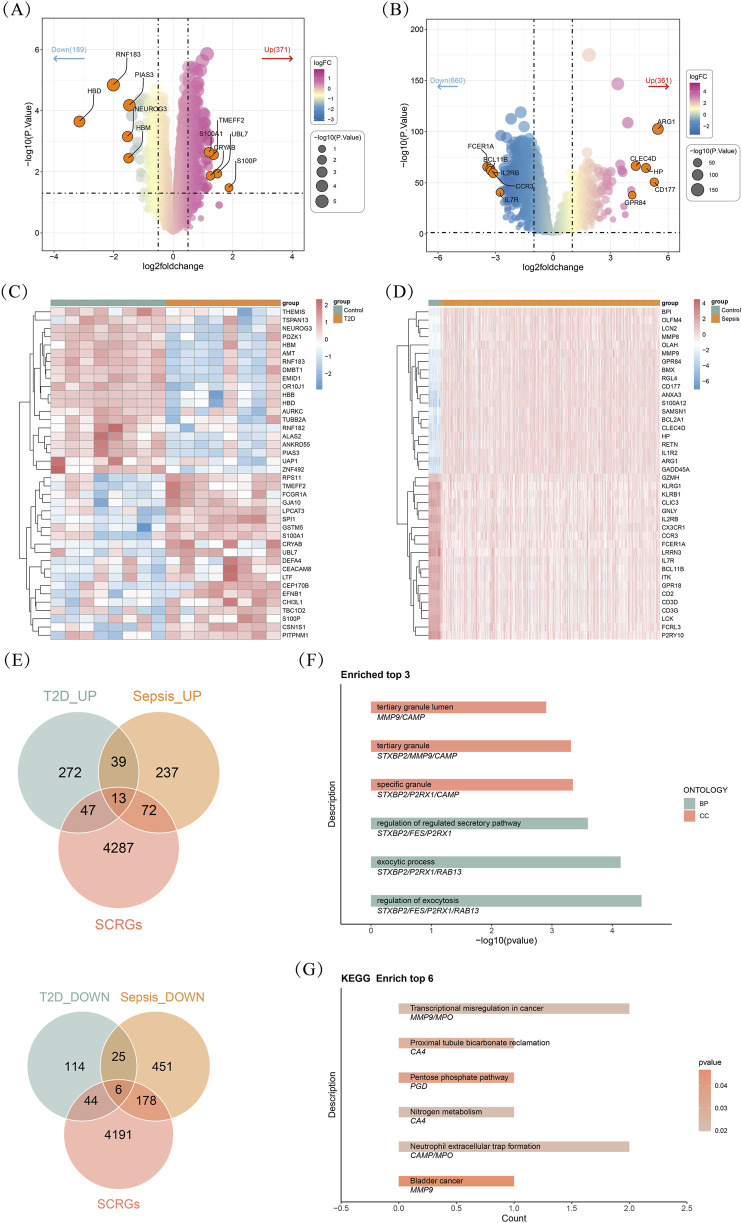
Identification and enrichment analysis of shared differentially expressed stem cell-related genes (DE-SCRGs) in type 2 diabetes mellitus (T2DM) and sepsis **(A)** The volcano plot showing the differentially expressed genes (DEGs) between T2DM and control groups **(B)** The volcano plot showing the DEGs between sepsis and control groups **(C,D)** The heatmaps showing the top 20 upregulated and downregulated genes **(C)** T2DM **(D)** Sepsis **(E)** The Venn diagram illustrating the intersection among DEGs between T2DM and controls, DEGs between sepsis and controls, and SCRGs **(F,G)** Functional Enrichment analyses for DE-SCRGs **(F)** Gene Ontology (GO) **(G)** Kyoto Encyclopedia of Genes and Genomes (KEGG).

Between T2DM and control groups, a total of 560 DEGs were identified, which 371 genes were upregulated and 189 genes were downregulated (Figure 1A). Similarly, the results of the comparison of the sepsis’s items discovered 921 DEGs, consisting of 261 upregulated and 660 downregulated genes (Figure 1B). As shown in Figures 1C,D, the top 20 signature in different trends in T2DM and sepsis were visualized, respectively. Following the intersection among DEGs in T2DM, DEGs in sepsis, and SCRGs, a total of 19 shared DE-SCRGs were obtained (upregulated items: 13; downregulated items: 6) (Figure 1E). Subsequently, GO and KEGG enrichment analyses were conducted to explore putative biological roles and channels of signaling. Results demonstrated that 20 GO items (17 GO BP items and three GO CC items) and 6 KEGG pathways were significantly enriched, such as “regulation of exocytosis (GO-BP),” “specific granule (GO-CC),” and “pentose phosphate pathway (KEGG)” (Figures 1F,G) (Supplementary Table S2).

### 3.2 Determination of stem cell-related biomarkers for T2DM and sepsis

**FIGURE 2:**
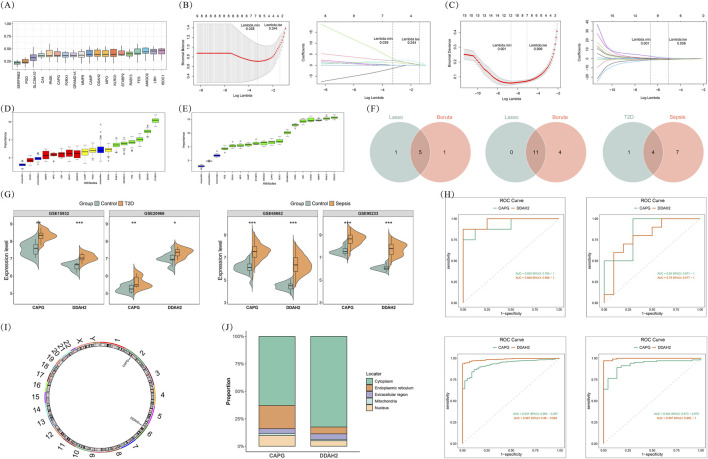
Determination of common stem cell-biomarkers for T2DM and sepsis **(A)** Functional similarity analysis of DE-SCRGs **(B,C)** Screening of characteristic genes through Least absolute shrinkage and selection operator (LASSO) regression **(B)** T2DM **(C)** Sepsis **(D,E)** Screening of characteristic genes through Boruta **(D)** T2DM **(E)** Sepsis **(F)** The Venn diagram illustrating the intersection among characteristic genes derived from two machine learning algorithms in T2DM and sepsis **(G)** Box plots revealing the differences in expression of candidate genes between T2DM and control groups and between sepsis and control groups From left to right: GSE15932; GSE20966; GSE65682; GSE95233 **(H)** The receiver operating characteristic (ROC) curve From left to right: GSE15932; GSE20966; GSE65682; GSE95233 **(I)** Chromosome localization analysis for biomarkers **(J)** Subcellular localization analysis for biomarkers.

To determine the common stem cell-related biomarkers for T2DM and sepsis, we first chose the top 15 genes based on functional similarity scores for the following analysis, namely, ISOC1, LBH, AMIGO2, FES, RAB13, STXBP2, KLRG1, MPO, DDAH2, CAMP, MMP9, GRAMD1A, P2RX1, CAPG, and PASK (Figure 2A). The LASSO analysis distinctly identified 6 characteristic genes for T2DM and 11 characteristic genes for sepsis (Figures 2B,C). Furthermore, the Boruta algorithm identified 6 characteristic genes for T2DM and 15 characteristic genes for sepsis, respectively (Figures 2D,E). The characteristic genes of two datasets were intersected to obtain four candidate genes, including RAB13, DDAH2, GRAMD1A, and CAPG (Figure 2F). Subsequently, expression analysis revealed that in both training and validation sets for T2DM and sepsis, CAPG and DDAH2 were significantly highly expressed in the disease group (Figure 2G). Moreover, ROC analysis suggested that the AUC values of these two genes in training and validation sets were all greater than 0.7, revealing that these two genes had good diagnostic efficacy for T2DM and sepsis (Figure 2H). Therefore, CAPG and DDAH2 were shared stem cell-related biomarkers for T2DM and sepsis. Chromosomal localization analysis demonstrated that CAPG was located on chromosome 2 and DDAH2 was located on chromosome 6 (Figure 2I). Subcellular localization analysis showed that the two biomarkers were mainly located in the cytoplasm (Figure 2J).

### 3.3 Development and evaluation of nomogram for T2DM and sepsis

**FIGURE 3:**
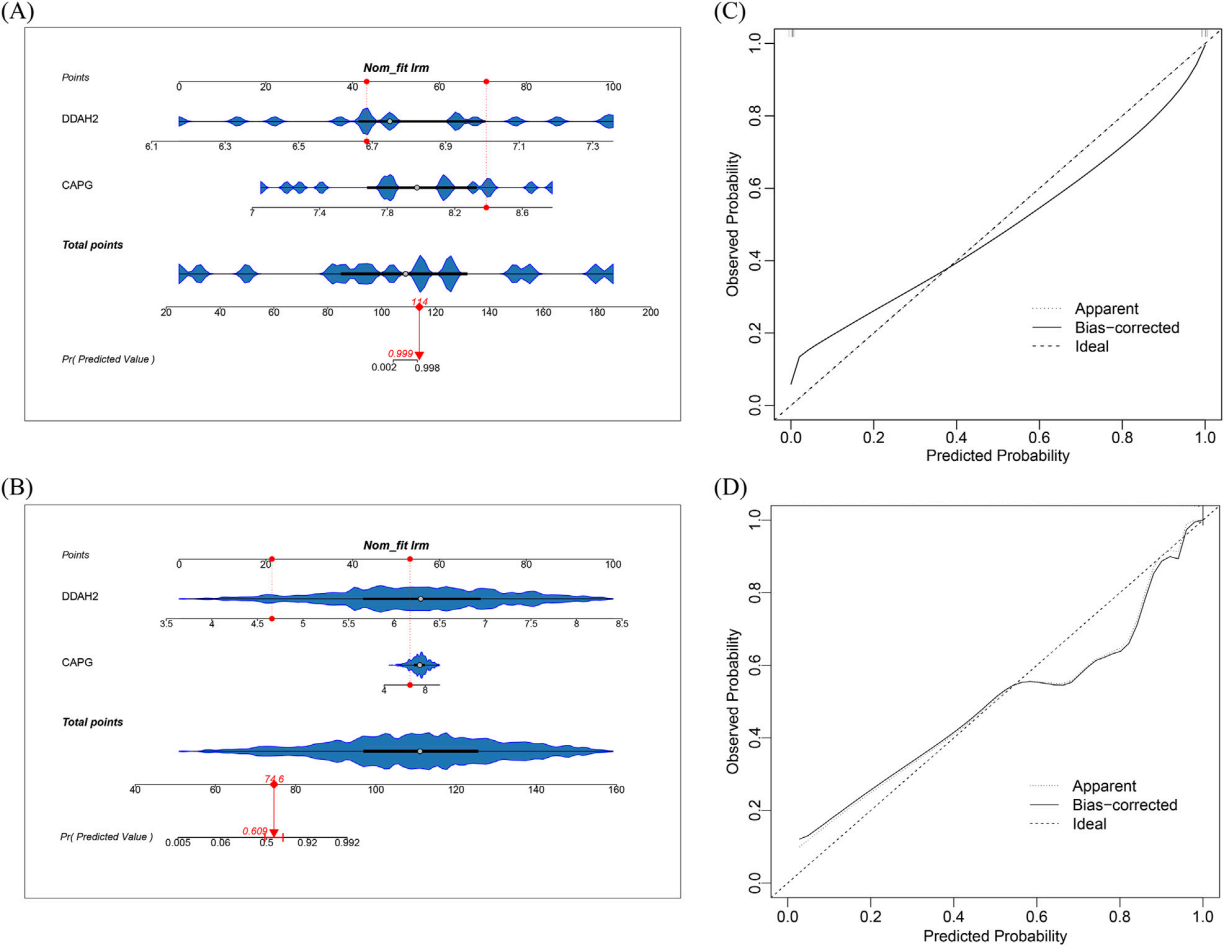
Development and assessment of nomogram **(A,B)** The nomogram **(A)** T2DM **(B)** Sepsis (Each dot represents the gene expression level and corresponding score within the sample, with the red line connecting the two.) **(C,D)** The calibration curve **(C)** T2DM **(D)** Sepsis.

To further determine the predictive potential of diagnostic biomarkers for T2DM and sepsis, the nomograms were created by considering the expression patterns of biomarkers (Figures 3A,B). In the nomogram, each gene expression is represented as a “point,” and the aggregate of the individual gene scores constitutes the “Total Points.” Moreover, calibration curves were produced to assess the prediction ability of the nomogram. For T2DM, the calibration curve exhibited a slope approximating 1 (Figure 3C). For sepsis, the same result was obtained (Figure 3D). These results illustrated the excellent predictive effectiveness of nomograms for T2DM and sepsis.

### 3.4 Functional enrichment analysis for biomarkers

**FIGURE 4:**
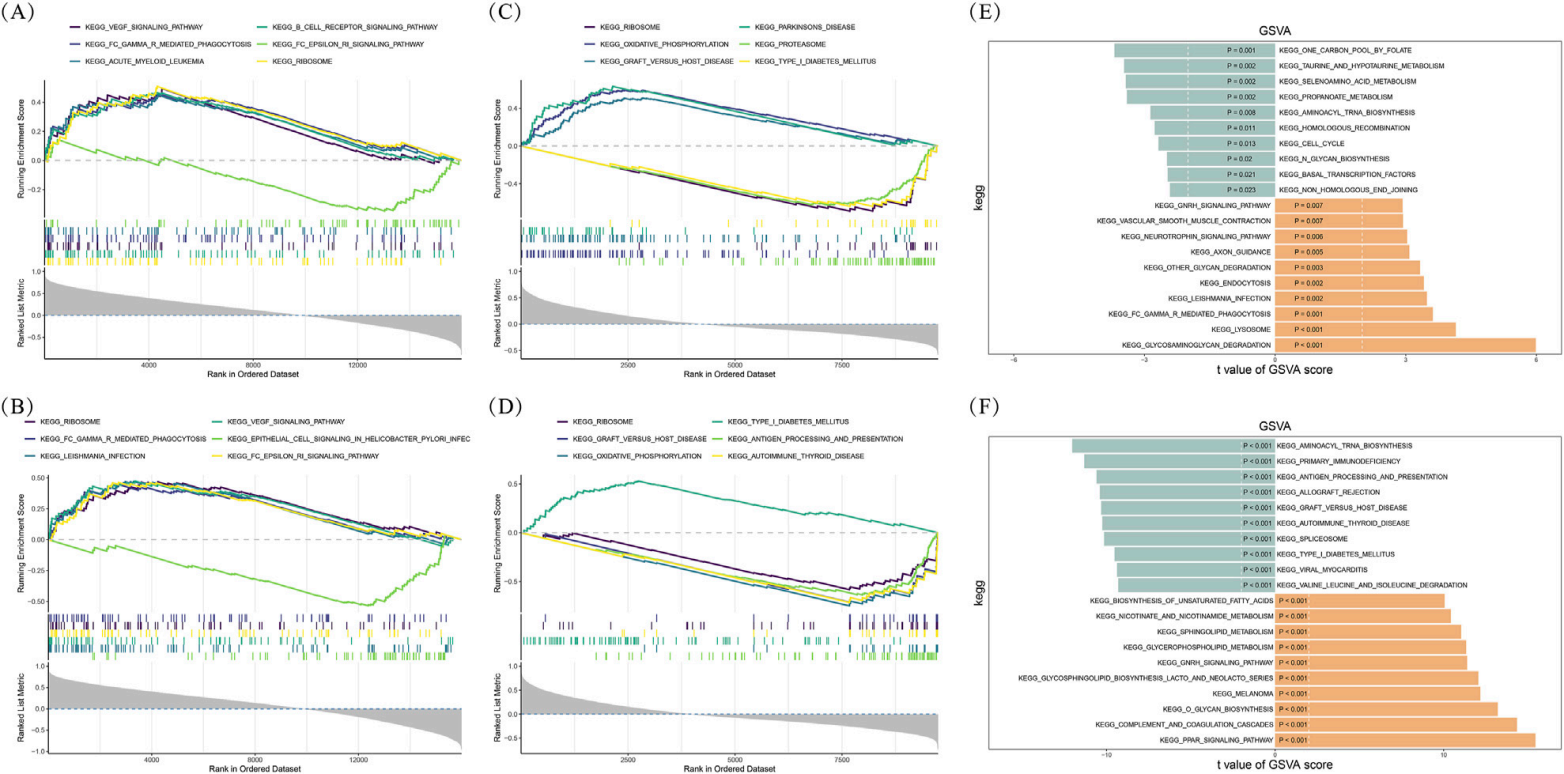
Gene set enrichment analysis (GSEA) and Gene set variation analysis (GSVA) **(A,B)** GSEA for biomarkers in T2DM **(A)** CAPG **(B)** DDAH2 **(C,D)** GSEA for biomarkers in sepsis **(C)** CAPG **(D)** DDAH2 **(E,F)** GSVA **(E)** T2DM **(F)** Sepsis.

To understand the functions of biomarkers, GSEA was conducted. Functional enrichment analysis using GO and KEGG databases revealed that CAPG and DDAH2 exhibit convergent roles in T2DM and sepsis. In T2DM (Figures 4A,B) (Supplementary Table S3), both genes were significantly enriched in immune-related pathways (e.g., FcγR-mediated phagocytosis, B cell receptor signaling), infectious disease pathways (e.g., *Helicobacter pylori* and Leishmania infections), and growth factor signaling (VEGF/ErbB pathways), and genetic information processing (translation)-related pathways (Ribosome).

In sepsis, CAPG and DDAH2 these genes showed broader functional repertoires, including Antigen processing and presentation, complement activation, and T cell receptor signalings. Disease-specific enrichments included autoimmune disorders (e.g., graft-versus-host disease), neurodegeneration (Parkinson’s disease), and metabolic reprogramming (oxidative phosphorylation) (Figures 4C,D) (Supplementary Table S4). Both diseases showed shared involvement in ribosomal machinery (Ribosome/Proteasome), revealing conserved roles in translational regulation of which in the development of diseases.

### 3.5 Identification of KEGG pathways associated with development of T2DM and sepsis

Furthermore, we also conducted GSVA to investigate KEGG pathways that were significantly relevant to the development of T2DM and sepsis. In T2DM, a total of 15 KEGG pathways were considerably suppressed, whereas 33 KEGG pathways were significantly activated (Figure 4E) (Supplementary Table S5). In sepsis, 55 KEGG pathways were considerably inhibited, and 108 KEGG pathways were markedly activated (Figure 4F) (Supplementary Table S5). After a comprehensive analysis, we found that a total of 11 KEGG pathways were inhibited in both T2DM and sepsis, including “aminoacyl tRNA biosynthesis,” “valine leucine and isoleucine degradation,” “pyruvate metabolism,” and eight other pathways (Supplementary Table S5). Additionally, 26 KEGG pathways were activated in both T2DM and sepsis, such as the ”GnRH/ErbB/neurotrophin signaling pathways,” among others (Supplementary Table S5). These results highlighted common molecular mechanisms that may contribute to the pathogenesis of T2DM and sepsis.

### 3.6 Investigation of immune cell infiltration and its correlation with biomarkers

**FIGURE 5:**
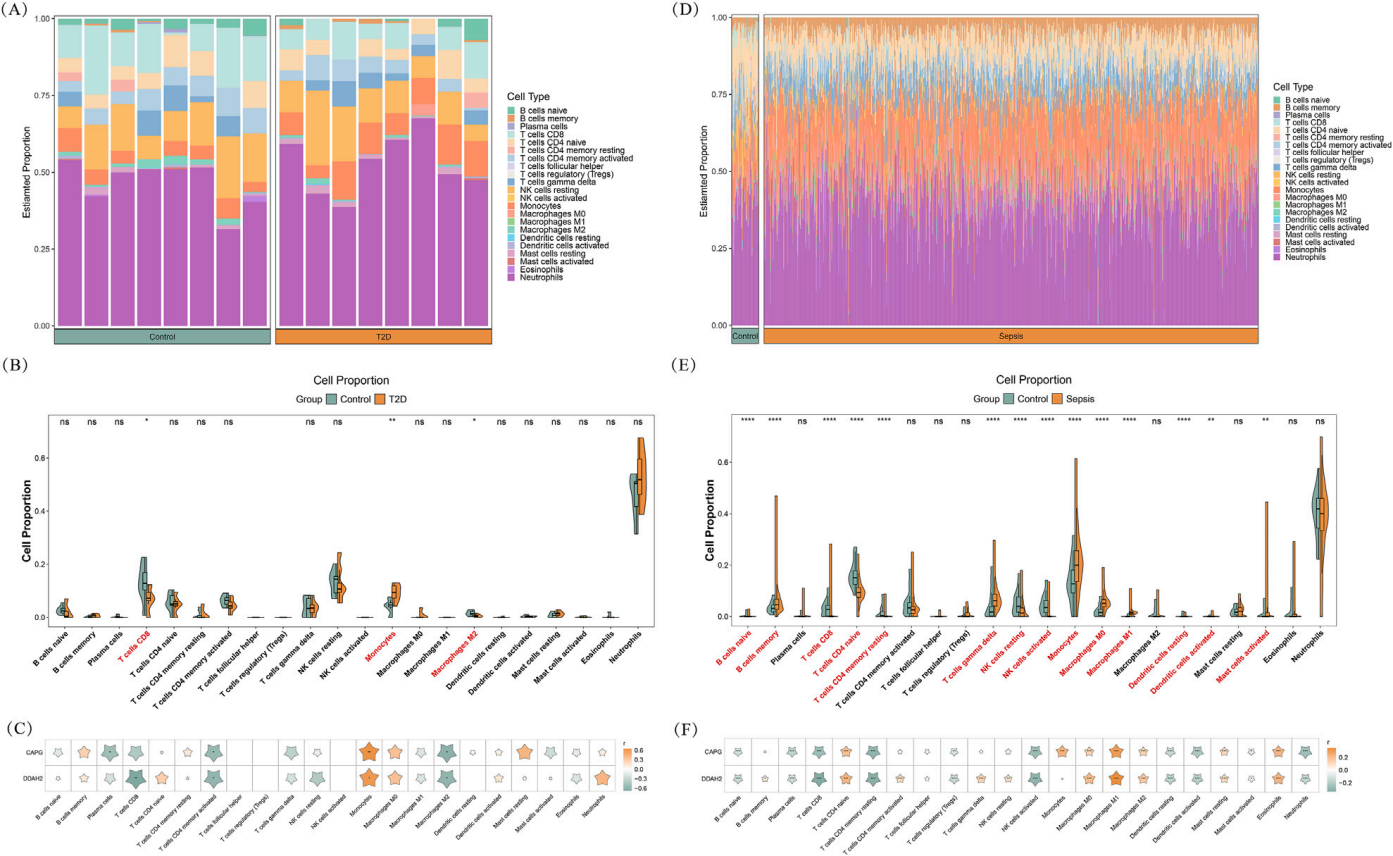
Immune Infiltration analysis **(A,C)** Immune infiltration analysis in T2DM **(A)** Stacked bar chart showing the infiltration abundance of each immune cell in each sample **(B)** Box plot revealing the differences in infiltration abundance of immune cells between T2DM and control groups **(C)** Heatmap illustrating correlations between biomarkers and immune cells **(D–F)** Immune infiltration analysis in sepsis **(D)** Stacked bar chart showing the infiltration abundance of each immune cell in each sample **(E)** Box plot revealing the differences in infiltration abundance of immune cells between sepsis and control groups **(F)** Heatmap illustrating correlations between biomarkers and immune cells ns: not significance *P < 0.05, **P < 0.01, ***P < 0.001, ****P < 0.0001.

Considering the important role of inflammation and immune response in sepsis and T2DM, the immune infiltration analysis was performed. As depicted in Figure 5A, the stacked bar chart demonstrated the relative abundance of immune cell subsets in each sample in GSE15932. Between T2DM and control groups, the infiltration abundance of CD8 T cells, monocytes, and M2 macrophages was markedly different (Figure 5B). Results of correlation analysis illustrated that two biomarkers had significant positive associations with monocytes and had marked negative relevance to activated memory CD4 T cells and M2 macrophages (Figure 5C). Furthermore, CAPG also enjoyed a substantial adverse relationship with plasma cells (cor = −0.509, *P* = 0.044), and DDAH2 had a marked negative relevance to CD8 T cells (cor = −0.679, *P* = 0.004) (Figure 5C).

Immune cell infiltration profiles of 22 subsets in sepsis and control samples was presented in Figure 5D. Comparative analysis revealed significant differences in 14 immune cell types, including CD8 T cells, naive B cells, resting natural killer (NK) cells, monocytes, M0 macrophages, resting dendritic cells, activated mast cells, and so on (Figure 5E). Correlation analysis demonstrated that, except for memory B cells, activated memory CD4 T cells, follicular helper T cells, gamma delta T cells, and resting NK cells, CAPG had significant correlations with the remaining immune cells, the strongest positive correlation was observed with M1 macrophages (cor = 0.305, *P* = 9.898e-19), while the highest negative correlation was with resting memory CD4 T cells (cor = −0.303, *P* = 1.652e-18) (Figure 5F). Except for follicular helper T cells and monocytes, DDAH2 had marked associations with remaining immune cells, with the highest positive correlation with macrophages M1 (cor = 0.349, *P* = 1.885e-24) and with the highest negative correlation with CD8 T cells (cor = −0.340, *P* = 4.035e-23) (Figure 5F).

### 3.7 Construction of biomarker interaction networks

**FIGURE 6:**
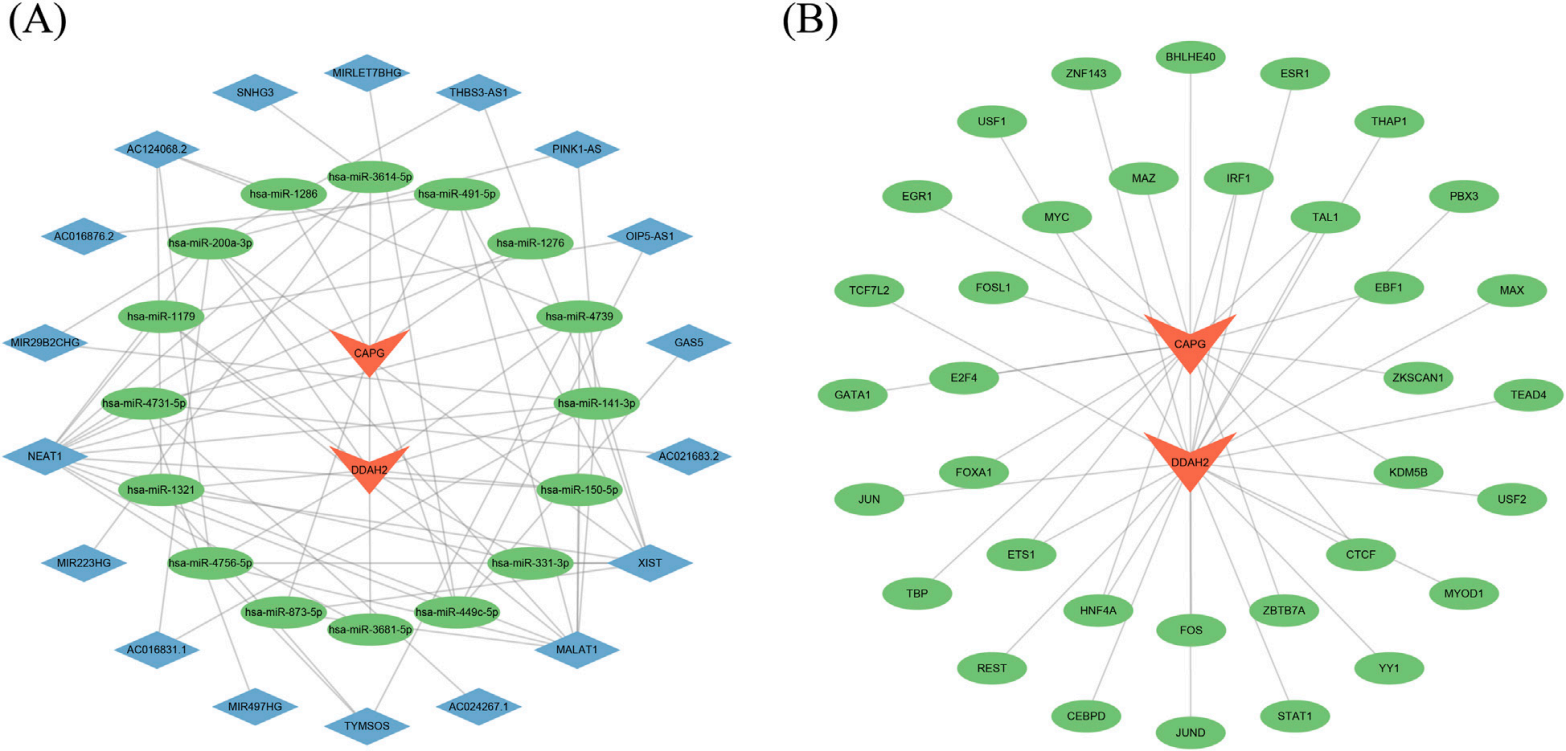
Construction of regulation networks **(A)** The lncRNA-miRNA-mRNA network Orange represents mRNAs, green represents miRNAs, and blue represents lncRNAs **(B)** The transcription factor (TF)-mRNAs Orange represents mRNAs and green represents TFs.

To confirm the upstream and downstream interactions of biomarkers and their related contents, we constructed the regulatory networks of biomarkers as well as the related networks of the drugs. Through the starbase database, a total of 7 miRNAs regulated CAPG, 11 miRNAs regulated DDAH2, and 68 miRNA-lncRNA relationships were predicted. After integration, the lncRNA-miRNA-mRNA regulator network was created, including 36 nodes (2 mRNAs, 16 miRNAs, and 18 lncRNAs) and 71 edges (Figure 6A). In which, the regulatory interactions comprised NEAT1-hsa-miR-1179-DDAH2 and NEAT1-hsa-miR-1276-CAPG. Through the ChEA3 database, 19 TFs for CAPG and 21 TFs for DDAH2 were predicted. After integration, the TF-mRNA regulator network was developed, consisting of 37 nodes (2 mRNAs and 35 TFs) and 40 edges (Figure 6B). Among these TFs, ETS1, CTCF, TAL1, HNF4A, and IRF1 all had a regulatory role for both CAPG and DDAH2. Results of drug prediction indicated that eight drugs had interactions with CAPG, and 25 drugs had interactions with DDAH2. The drug-mRNA network was comprised of 30 edges and 33 edges (Supplementary Figure S1A). Subsequently, according to the *P*-value ranking, we selected the top 2 drugs for molecular docking. Results revealed that puromycin interacted with CAPG by hydrophobic bonds to ARG-112, GLY-113, TYR-109, GLN-39, and GLN-36 (Supplementary Figure S1B). Retinoic acid engaged with CAPG through hydrophobic interactions with TRP-157 (Supplementary Figure S1C). Nebivolol collaborates with DDAH2 by hydrophobic links to ARG-173, MET-178, and PHE-222 (Supplementary Figure S1D). Podophyllotoxin interacted with DDAH2 by hydrophobic bonds to ARG-10 and PHE-222 (Supplementary Figure S1E). Moreover, the binding energies were all less than −5 kcal/mol, suggesting that the bonds were all stable (Supplementary Table S6).

### 3.8 Analysis of expression level of biomarkers at the cellular level in T2DM

**FIGURE 7:**
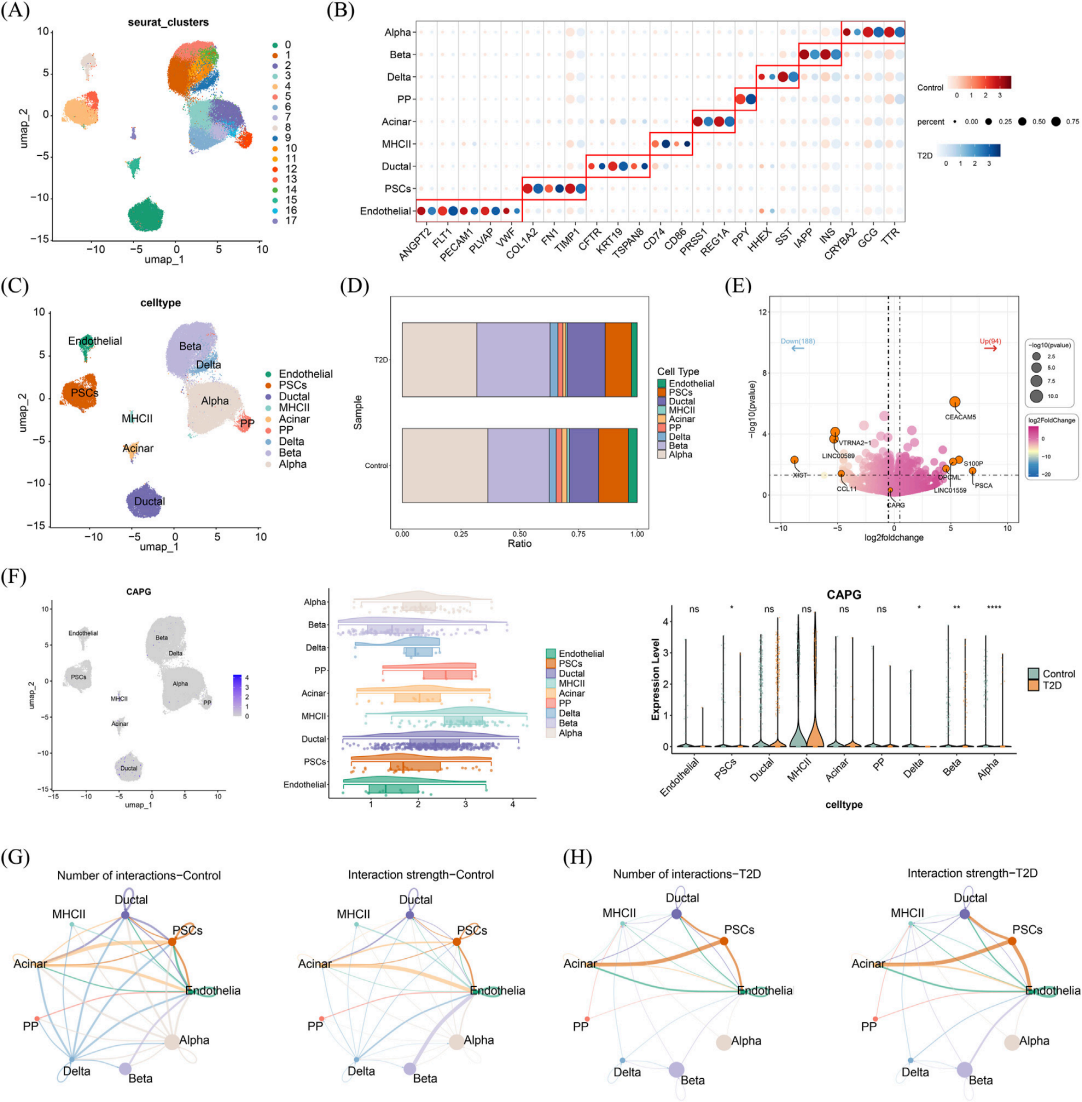
The expression of biomarkers in the cellular level **(A)** UMAP showing 18 different clusters **(B)** Bubble diagram shows the expression of marker genes in each annotated cell types **(C)** UMAP distribution of 9 cell types **(D)** The proportions of each cell type in control and T2DM groups **(E)** The volcano plot showing the DEGs of the RNA-seq dataset **(F)** The expression of each biomarker in each cell type **(G)** Cell communication networks illustrating the number and strength of interactions among cell types in the control group **(H)** Cell communication networks illustrating the number and strength of interactions among cell types in the T2DM groups ns: not significance *P < 0.05, **P < 0.01, ***P < 0.001, ****P < 0.0001.

To further evaluate the expression of biomarkers at the cellular level, single-cell analysis was carried out. After QC, remaining high-quality cell populations were clustered into 18 clusters, which were annotated with 9 cell types: acinar cells, alpha cells, beta cells, delta cells, ductal cells, endothelial cells, antigen-presenting MHC class II cells (MHC II), pancreatic polypeptide (PP) cells, and pancreatic stellate cells (PSCs) (Figures 7A–C; Supplementary Figure S2). In T2DM and control samples, the 2 cell types with the largest proportion were alpha cells and beta cells (Figure 7D). DDAH2 was not detected in the scRNA-seq dataset due to the absence of information for this gene in the original data. Therefore, subsequent single-cell analyses focused only on CAPG (Figure 7E). Results demonstrated that CAPG was expressed in all cell types, and the expression in alpha cells, beta cells, delta cells, and PSCs between T2DM and control groups was significantly different (Figure 7F).

Further analysis of pancreatic cell–cell communication revealed that, in the context of T2DM, interactions between PSCs and endothelial cells, as well as between endothelial cells and other pancreatic cell types, were more pronounced compared with controls (Figures 7G,H). Specifically, in T2DM, PSCs established additional connections with endothelial cells through VEGFA–FLT1 and ECM–integrin axes, whereas in controls, EC-to-PSC interactions were mainly mediated by ANGPTL4–SDC2/CDH11. Beyond PSC–EC crosstalk, ductal cells in T2DM lost their interactions with PSCs but gained connections with MHC II cells; acinar cells only interacted with endothelial cells; PP cells established additional interactions with MHC II cells; delta cells interacted with MHC II, endothelial, and beta cells; and alpha cells gained interactions with MHC II and endothelial cells. By contrast, the connection between beta cells and endothelial cells was diminished in T2DM (Supplementary Table S7). As no blood-based scRNA-seq datasets with case–control design were available, all single-cell analyses and cell–cell communication results—including those from CellChat—were pancreas-specific and limited to the T2DM context. These findings should not be interpreted as reflective of systemic immune processes or sepsis-related interactions.

### 3.9 Validation of expression of biomarkers

**FIGURE 8:**
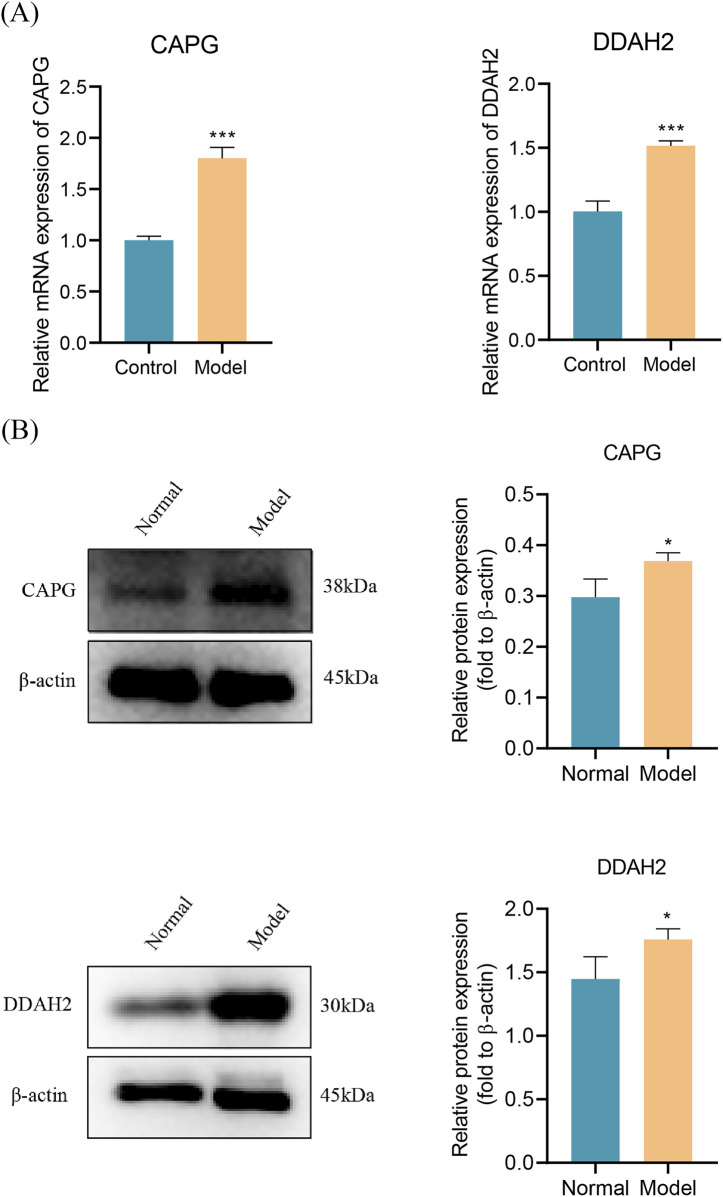
The validation of expression of biomarkers **(A)** The expression of biomarkers at the transcription (mRNA) level (n = 3) **(B)** The expression of biomarkers at the protein level (n = 3). From left to right: CAPG, DDAH2. *P < 0.05, **P < 0.01, ***P < 0.001, ****P < 0.0001.

To further validate the expression of biomarkers, we developed an *in vitro* model of T2DM-sepsis. Quantitative analysis revealed that both CAPG and DDAH2 exhibited significantly higher mRNA and protein expression in the model group compared to the control group (Figures 8A,B). All experiments, including qPCR and Western blot, were independently repeated three times, and the results were statistically significant. These findings are consistent with the bioinformatics analysis and further support the potential of CAPG and DDAH2 as diagnostic biomarkers for T2DM and sepsis.

## 4 Discussion

The intersection of T2DM and sepsis is significant at the clinical and molecular levels, and Mendelian randomization studies have further confirmed that patients with T2DM have a significantly increased risk of developing sepsis (Qi et al., 2025). This study identifies, for the initial time, the stem cell-associated molecular network common to T2DM and sepsis, which provides a new perspective to understand the mechanism behind their co-morbidity. CAPG and DDAH2, as key node genes, not only serve as early diagnostic biomarkers but also possibly influence diseases by the immune-metabolism pathway, proving a theoretical foundation for the development of stem cell-based combination therapies in T2DM and sepsis.

CAPG, as an actin-binding protein, has been predominantly studied for its roles in tumor metastasis and migration of immune cells. Glaser et al. established that CAPG plays a role in the spreading and invasiveness of ovarian carcinoma (Glaser et al., 2014). CAPG has been shown to facilitate gastric cancer proliferation, migration, invasion, and metastasis in both *in vivo* and *in vitro* models (Long et al., 2024). In colorectal cancer, mechanistic studies reveal that CAPG exerts oncogenic functioms by inhibiting apoptosis and ferroptosis, while promoting colorectal cancer cell proliferation through repression of the P53 pathway (Zhao et al., 2023). In our study, CAPG showed significant correlations with monocytes and macrophages in both T2DM and sepsis, revealing that it may be involved in T2DM and sepsis by affecting monocyte/macrophage functions, such as phagocytosis and chemotaxis, thereby broadening its pathophysiological role. In T2DM, M1 macrophage polarization exacerbates adipose tissue inflammation and insulin resistance (Phu et al., 2022), whereas high expression of CAPG may amplify this process by enhancing the migration of monocytes to inflammatory sites. In sepsis, the overactivation of M1 macrophages is an important driver of the “cytokine storm” (Yan et al., 2024) with CAPG potentially contributing to the immune imbalance by regulating macrophage phagocytosis. DDAH2, a key enzyme in the nitric oxide (NO) metabolic pathway, plays critical physiological roles by degrading asymmetric dimethylarginine (ADMA), an exogenous modulator of nitric oxide synthase (NOS) (Liu et al., 2012). Plasma ADMA levels are elevated (Abhary et al., 2010) and DDAH expression and activity are reduced in diabetic patients (Yuan et al., 2015). However, there are differences in the effect of DDAH gene polymorphisms on serum ADMA levels in type 1 diabetes mellitus (T1DM) and T2DM (Fogarty et al., 2012). Research utilizing animal models has indicated that abnormalities in adipose tissue ADMA metabolism may correlate with mechanisms governing blood flow in white adipose tissue in GK rats, a model for T2DM. In sepsis pathophysiology, the concentration of ADMA in the plasma increases (Weiss et al., 2012), and the expression and activity of DDAH2 in immune cells decrease (Winkler et al., 2017). This may not only lead to the passivation of NO signals but also result in the subsequent impairment of pathogen defense, indicating that DDAH2 promotes the response to severe bacterial sepsis to a large extent by regulating the inflammatory response of macrophages (Lambden et al., 2015; Lambden et al., 2018; Winkler et al., 2017). Furthermore, studies had been demonstrated that plasma ADMA can be used as a biomarker for the prognosis of septic shock (Lambden et al., 2018). Notably, the negative correlation between DDAH2 and CD8^+^ T cells was significant in both diseases, possibly reflecting its suppression of T cell toxicity through NO signaling (Huang et al., 2021a), a mechanism that may have a double-edged effect in the autoimmune injury of T2DM and the immunosuppressive phase of sepsis. Despite the absence of DDAH2 expression in pancreatic single-cell data—which is consistent with previously published single-cell datasets showing that DDAH2 is almost undetectable and expressed at extremely low levels in normal islet cells (Baron et al., 2016; Segerstolpe et al., 2016)—DDAH2 exhibited consistent upregulation in bulk transcriptomic analyses and in our *in vitro* model. Under combined high-glucose and LPS stimulation, designed to mimic the inflammatory and metabolic stress of T2DM complicated with sepsis, its expression was markedly induced, supporting the role of DDAH2 as an inducible marker of systemic immune stress in T2DM and sepsis. In summary, these findings highlight the complex roles of CAPG and DDAH2 in modulating immune cell functions in both T2DM and sepsis, suggesting that targeting these proteins may offer potential therapeutic strategies for managing inflammation and immune dysregulation in these diseases.

The GSEA findings indicated that CAPG and DDAH2 were strongly implicated in “FC gamma R-mediated phagocytosis” and “Ribosome” in both T2DM and sepsis, further indicating they share the same mechanism of immunometabolic reprogramming. The FC gamma R-mediated phagocytosis is a key hub connecting innate immunity and adaptive immunity, and its abnormal activation is associated with multiple immune dysfunctions. Enrichment of the Ribosome suggests a compensatory enhancement of protein synthesis under cellular stress, a phenomenon that may occur in both pancreatic β-cells in T2DM (in response to insulin secretion load) and immune cells in sepsis (in response to pathogen attack). In addition, GSVA results showed activation of the ErbB signaling pathway is significant in both diseases. This pathway may be involved in islet compensatory hyperplasia (T2DM) (Arai et al., 2025) and post-sepsis tissue repair by regulating cell proliferation and survival (Zhang et al., 2022), a common mechanism that provides clues for the development of broad-spectrum therapies. The findings systematically elucidate the molecular similarities between T2DM and sepsis concerning immune-metabolic regulation, formulating a theoretical foundation for the advancement of synergistic methods for therapy that concurrently target multiple immune-metabolic pathways.

Finally, we also created the lncRNA-miRNA-mRNA, TF-mRNA, and drug-mRNA networks in this study to elucidate the complex regulatory interactions and potential therapeutic targets underlying the shared pathophysiology of T2DM and sepsis. In the lncRNA-miRNA-mRNA network, NEAT1 regulates CAPG/DDAH2 through miR-1179/miR-1276, which is consistent with previously reported pivotal roles of NEAT1 in inflammation and metabolic diseases (Jia et al., 2022; Wang et al., 2018). Co-regulation of the transcription factors ETS1 and IRF1 suggests that CAPG/DDAH2 may be upregulated in response to inflammatory signals (e.g., TNF-α/IL-6) (Huang et al., 2021b; Meerson et al., 2013; Sacks et al., 2018). Among the drug predictions, puromycin (targeting CAPG) and nebivolol (targeting DDAH2) were identified as potential candidates, supported by favorable binding affinities (binding energy < −5 kcal/mol) based on molecular docking results. Nebivolol, a β-receptor blocker with known cardiovascular protective effects (Kadi et al., 2008), has also been proposed to improve endothelial function in sepsis-related contexts (Girardis et al., 2024), suggesting possible avenues for drug repurposing in the treatment of T2DM and sepsis.

In this study, we identified two shared stem cell-related biomarkers (CAPG and DDAH2) for T2DM and sepsis and further validated the expression of biomarkers through *in vitro* cell experiments. A developed nomogram based on biomarkers showed high predictive accuracy and clinical applicability. However, as the existing analysis is based on public databases and lacks validation with multicenter clinical samples, and the mechanistic depth is insufficient, the specific role of CAPG/DDAH2 in immune-inflammation has not been verified by knockout/overexpression experiments. In addition, validation partly relied on pancreatic scRNA-seq due to limited public datasets. While signals were consistent across cohorts, pancreatic profiles may not reflect blood or systemic patterns. The lack of suitable blood scRNA-seq data highlights the need for future studies using peripheral blood or cross-tissue transcriptomics to confirm these biomarkers. Further exploration is still needed in the future.

## 5 Conclusion

This study determines shared stem cell-related biomarkers (CAPG and DDAH2) for T2DM and sepsis through bioinformatics analysis and cell experiments and finds that these biomarkers may modulate diseases through immune-inflammation. These discoveries have enhanced our comprehension of the inherent connection between T2DM and sepsis, offering novel insights into the diagnosis and management of the condition.

## Data Availability

The datasets (GSE15932, GSE20966, GSE65682, GSE95233, and GSE195986) analyzed in this study were collected from the Gene Expression Omnibus (GEO) database (https://www.ncbi.nlm.nih.gov/geo).
