# Supplementary material for: Integrated analysis of stem cell-related genes shared between type 2 diabetes mellitus and sepsis
Source: Front Chem. 2025 Sep 19;13:1666651. doi: 10.3389/fchem.2025.1666651 (PMC12491291; doi:10.3389/fchem.2025.1666651)
Supplement: Supplementary file 5 [file Table6.docx]

**Supplementary Table 6** Molecular docking between biomarkers and drugs

| Gene | UniProt | Drug name | CID | Binding energy | Number of hydrogen bonds |
| --- | --- | --- | --- | --- | --- |
| CAPG | B8ZZL6 | puromycin | CID439530 | -10.05 | 7 |
| CAPG | B8ZZL6 | Retinoic acid | CID444795 | -8.41 | 1 |
| DDAH2 | O95865 | Nebivolol | CID71301 | -11.28 | 2 |
| DDAH2 | O95865 | podophyllotoxin | CID10607 | -13.37 | 3 |
