# Supplementary material for: Integrated analysis of stem cell-related genes shared between type 2 diabetes mellitus and sepsis
Source: Front Chem. 2025 Sep 19;13:1666651. doi: 10.3389/fchem.2025.1666651 (PMC12491291; doi:10.3389/fchem.2025.1666651)
Supplement: Supplementary file 8 [file DataSheet1.docx]

Supplementary Material

# Supplementary Figures and Tables

## Supplementary Figures


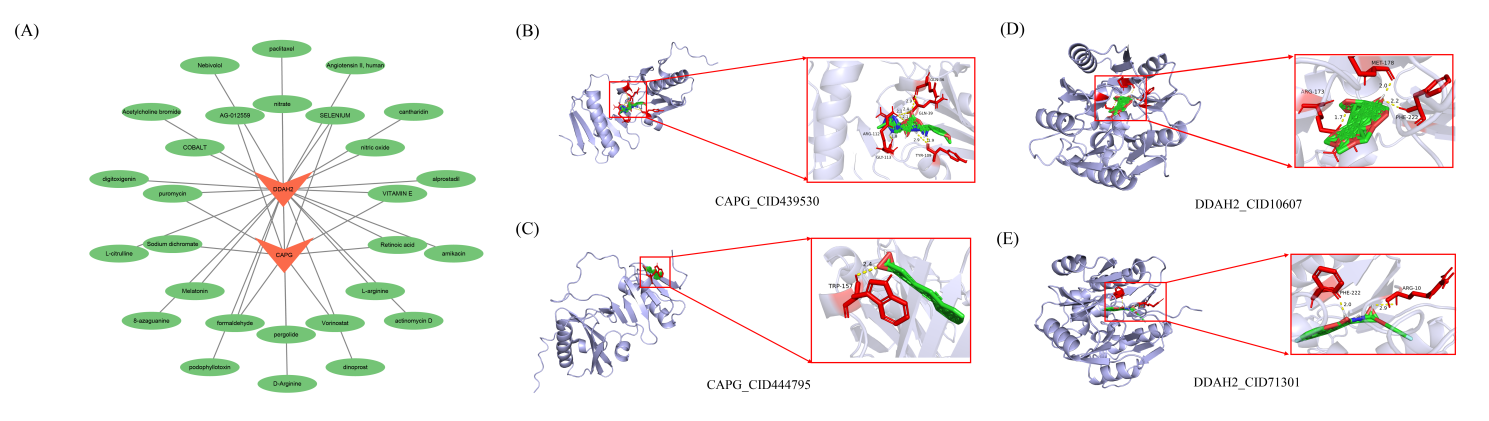


**Supplementary Figure 1.** Drug prediction analysis (**A**) The drug-mRNA network Orange represents mRNAs and green represents drugs (**B**) Molecular docking between CAPG and puromycin (**C**) Molecular docking between CAPG and retinoic acid (**D**) Molecular docking between DDAH2 and nebivolol (**E**) Molecular docking between DDAH2 and podophyllotoxin


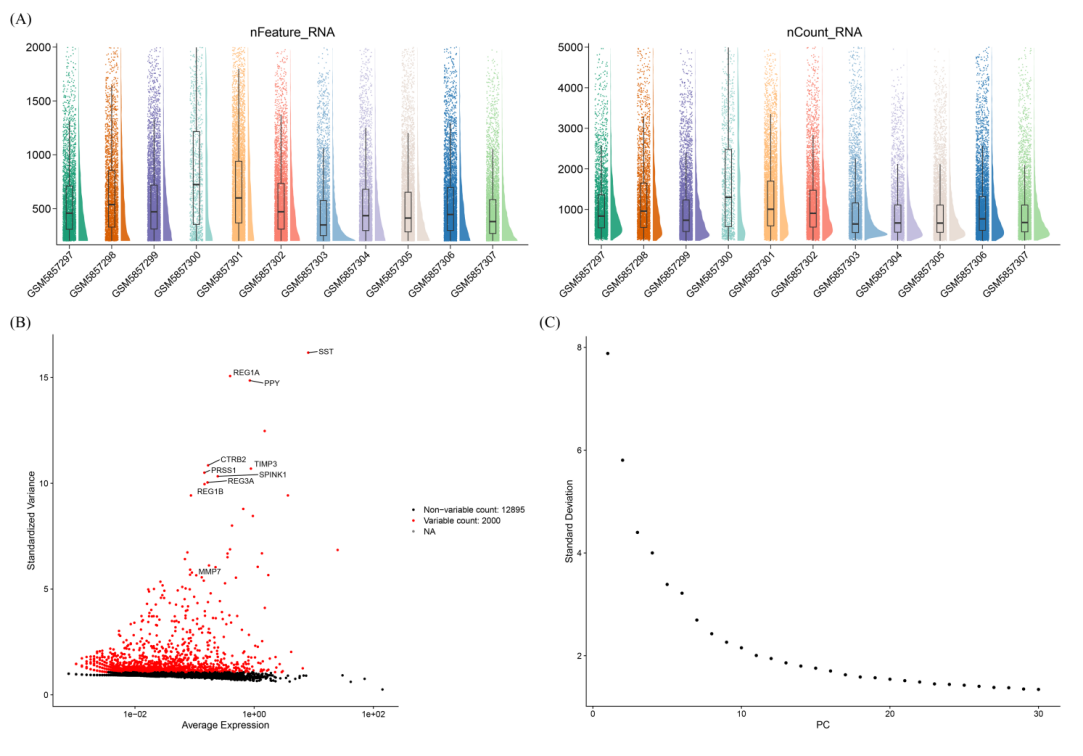


**Supplementary Figure 2.** Quality control (QC) of single-cell dataset (**A**) Distribution of cells in each sample after QC in terms of nFeature_RNA and nCount_RNA (**B**) Screening of 2,000 highly variable genes (**C**) principal component analysis (PCA) dimensionality reduction analysis

## Supplementary Tables

**Supplementary Table 1** DEG Profiles from GSE15932 and GSE65682 Datasets.

**Supplementary Table 2** List of GO items and KEGG pathways that DE-SCRGs enriched.

**Supplementary Table 3** List of KEGG pathways enriched of biomarkers by GSEA in T2DM.

**Supplementary Table 4** List of KEGG pathways enriched of biomarkers by GSEA in sepsis.

**Supplementary Table 5** List of KEGG pathways enriched by GSVA in T2DM and sepsis.

**Supplementary Table 6** Molecular docking between biomarkers and drugs.

**Supplementary Table 7** Results of cell–cell communication analysis.
